# Disruption of CRTC1 and CRTC2 in Sim1 cells strongly increases high-fat diet intake in female mice but has a modest impact on male mice

**DOI:** 10.1371/journal.pone.0262577

**Published:** 2022-01-12

**Authors:** Jin Tanaka, Fuka Ishikawa, Tomoki Jinno, Motoki Miyakita, Haruka Miyamori, Tsutomu Sasaki, Takumi Yokokawa, Tsuyoshi Goto, Kazuo Inoue, Shigenobu Matsumura

**Affiliations:** 1 Division of Food Science and Biotechnology, Graduate School of Agriculture, Kyoto University, Kyoto, Japan; 2 Department of Neurology, Graduate School of Medicine, Osaka University, Osaka, Japan; 3 Department of Clinical Nutrition, Graduate School of Comprehensive Rehabilitation, Osaka Prefecture University, Osaka, Japan; Tokyo University of Agriculture, JAPAN

## Abstract

cAMP responsive element binding protein (CREB)-regulated transcription coactivators (CRTCs) regulate gene transcription in response to an increase in intracellular cAMP or Ca^2+^ levels. To date, three isoforms of CRTC have been identified in mammals. All CRTCs are widely expressed in various regions of the brain. Numerous studies have shown the importance of CREB and CRTC in energy homeostasis. In the brain, the paraventricular nucleus of the hypothalamus (PVH) plays a critical role in energy metabolism, and CRTC1 and CRTC2 are highly expressed in PVH neuronal cells. The single-minded homolog 1 gene (Sim1) is densely expressed in PVH neurons and in some areas of the amygdala neurons. To determine the role of CRTCs in PVH on energy metabolism, we generated mice that lacked CRTC1 and CRTC2 in Sim1 cells using Sim-1 cre mice. We found that Sim1 cell-specific CRTC1 and CRTC2 double-knockout mice were sensitive to high-fat diet (HFD)-induced obesity. Sim1 cell-specific CRTC1 and CRTC2 double knockout mice showed hyperphagia specifically for the HFD, but not for the normal chow diet, increased fat mass, and no change in energy expenditure. Interestingly, these phenotypes were stronger in female mice than in male mice, and a weak phenotype was observed in the normal chow diet. The lack of CRTC1 and CRTC2 in Sim1 cells changed the mRNA levels of some neuropeptides that regulate energy metabolism in female mice fed an HFD. Taken together, our findings suggest that CRTCs in Sim1 cells regulate gene expression and suppress excessive fat intake, especially in female mice.

## Introduction

The hypothalamus plays an important role in the energy metabolism. Several genes that regulate appetite and energy expenditure in neuronal cells are tightly regulated by endogenous or exogenous factors. Therefore, dysregulation of gene expression causes obesity [1,2]. Various transcription factors regulate gene expression in neuronal cells. Among these transcription factors, cAMP responsive element binding protein (CREB) plays an important role in regulating neuronal function and neuropeptide expression, affecting whole-body energy metabolism [3,4]. In addition to CREB, recent studies have highlighted the importance of CREB-regulated transcription coactivator (CRTC) in various cell types [5,6]. Three isoforms of CRTC have been identified in rodents and humans [7]. Under basal conditions, all isoforms of CRTC are phosphorylated by salt-inducible kinases, likely cooperating with other kinases, and sequestered in the cytoplasm by association with 14-3-3 proteins [8]. Triggering of the cyclic adenosine monophosphate (cAMP) pathway promotes the inhibitory phosphorylation of salt-inducible kinases by cAMP-dependent protein kinase (PKA), and Ca^2+^ influx activates calcineurin, which can dephosphorylate CRTCs. These pathways lead to the dephosphorylation and translocation of CRTCs to the nucleus, where they co-activate target genes via interactions with CREB and CREB-binding proteins [9,10].

All isoforms of CRTC were found in the neurons of the hypothalamus [11]. Within the hypothalamus, the paraventricular hypothalamic nucleus plays a pivotal role in regulating appetite and energy expenditure [12]. Single-minded homolog-1 (Sim1) is a transcription factor involved in the development of PVH neurons. Sim1 haplodeficiency or postnatal deficiency of Sim1 causes early onset obesity with hyperphagia [13,14]. Various neuropeptides such as corticotropin-releasing factor (CRF), arginine vasopressin (AVP), oxytocin (Oxt), and pituitary adenylate cyclase-activating polypeptide (PACAP) are expressed in PVH and regulate energy metabolism [15]. Neuropeptide receptors are also found in PVH [16,17]. The melanocortin 4 receptor (MC4R) in the PVH is one of the most important receptors regulating appetite [18]. A previous study has reported that MC4R null mice show hyperphagia and reduced energy expenditure, but rescue of the MC4R gene in PVH only suppressed hyperphagia in MC4R null mice [19]. MC4R couples to Gs, which increases cAMP levels after ligand binding [20]. It is likely that the increase in cAMP by MC4R ligand binding may activate CREB and CRTCs.

We previously investigated whether CRTC1 deficiency in PVH (Sim1 cells) could cause obesity. However, Sim1 cells specific CRTC1 knockout mice only showed a slight increase in body weight in HFD mice compared to wild-type mice [21]. In addition to CRTC1, CRTC2 is expressed in PVH, and low expression of CRTC3 is found in PVH [11]. Since CRTC2 whole body knockout mice showed a lean phenotype even in the HFD fed condition, it is likely that the deletion of CRTC2 in PVH would not cause hyperphagia and obesity [22]. However, we expected that the compensatory action of CRTC2 might cancel the effect of CRTC1 deficiency in PVH on body weight gain. To block the compensatory action of CRTC2, in the present study, we generated mice that lacked both CRTC1 and CRTC2 specifically in the PVH and evaluated the role of CRTCs in PVH in energy metabolism.

## Materials and methods

### Animals

The present study was conducted in accordance with the ethical guidelines of the Kyoto University Animal Experimentation Committee and the ethical guidelines of the Endocrine Society. All procedures were performed in compliance with the National Institute of Health Guide for the Care and Use of Laboratory Animals, and the Kyoto University Animal Care and Use Committee approved all procedures (approval number: 30–44). Every effort was made to minimize the number of animals used and limit experimentation, which was necessary to produce reliable scientific information, and all efforts were made to minimize suffering. At the end of the experimental period, mice were euthanized by deep isoflurane anesthesia. For tissue collection, mice were euthanized by pentobarbital administration (150 mg/kg) followed by cervical dislocation.

Sim1 cell-specific CRTC1 and CRTC2 double knockout mice (Sim1-CRTCDKO) were generated by crossing *Crtc1*^loxP/loxP^ mice [21], *Crtc2*^loxP/loxP^ mice [23], and *single-minded homolog-1* Cre (*Sim1*-cre) mice [19]. *Crtc2*^loxP/loxP^ mice were kindly provided by Prof. Klaus H. Kaestner (University of Pennsylvania, Philadelphia, PA, USA), and *Sim1*-cre mice were kindly provided by Prof. Joel Elmquist (UT Southwestern Medical Center, Dallas, TX, USA). Before mating, *Crtc1*^loxP/loxP^, *Crtc2*^loxP/loxP^, and *Sim1*-cre mice were backcrossed with C57BL/6 mice at least seven times. *Crtc1*^loxP/loxP^; *Crtc2*^loxP/loxP^; *Sim1*-cre^+^ (Sim1-CRTCDKO) and *Crtc1*^loxP/loxP^; *Crtc2*^loxP/loxP^; *Sim1*-cre^-^ (control) mice were used in all experiments.

The mice were housed 3–6 per cage in a vivarium maintained at 23 ± 2°C under a 12:12 h light/dark cycle (lights on from 0600 to 1800 h). For measurement of food intake and pair-feeding experiments, mice were housed in individual cages. Commercially available normal laboratory chow diet (NCD; Oriental Yeast, Tokyo, Japan) and water were available ad libitum. In the experiments, we used a high-fat diet (HFD; D12492; Research Diet, New Brunswich, NJ, USA), AIN-93M diet (Oriental Yeast, Tokyo, Japan), 20% sucrose mixed diet (SD), and 20% lard mixed diet (LD). To generate SD and LD, caster sugar (Mitsui Sugar, Tokyo, Japan) or Lard (Megmilk Snow Brand, Sapporo, Japan) were mixed in an AIN-93G diet (Oriental Yeast, Tokyo, Japan). Unless otherwise described, the NCD was switched to HFD at six weeks of age in the HFD feeding experiments. The composition and caloric density of the diets are presented in Table 1.

**Table 1:** Composition and caloric density of the diets.

	NC	AIN-93M	HFD	SD	LD
Protein (kcal%)	26	15	20	16	13
Fat (kcal%)	13	9	60	13	46
Carbohydrate (kcal%)	61	76	20	71	41
Caloric density (kcal/g)	3.60	3.85	5.24	3.97	5.00

### Immunohistochemistry

Mice were anesthetized with sodium pentobarbital (100 mg/kg) and perfused via the aorta with phosphate buffered saline (PBS) followed by 4% paraformaldehyde. Brains were removed and fixed in 4% paraformaldehyde. Samples were sliced into 30 μm-thick sections and placed in PBS. After preincubation with Block Ace (Yukijirushi, Sapporo, Japan), the sections were incubated overnight with rabbit anti-CRTC1 antibody (#2587, 1: 1000 dilution, Cell Signaling Technology, Danvers, MA, USA) or anti-CRTC2 antibody (#3826, 1: 1000 dilution, Cell Signaling Technology) at room temperature (20–30°C). After several rinses in PBS, the sections were incubated with Alexa Fluor 555-conjugated donkey anti-rabbit IgG (# A-31572, 1: 500 dilution, Thermo Fisher Inc., San Jose, CA). DAPI was used for nuclear staining. Immunostained sections were observed under a confocal laser scanning microscope.

### Respiratory gas analysis

After 4 weeks of feeding with the HFD, the mice were held individually in a chamber for 48 h to attain a constant respiratory exchange ratio (RER) (n = 6). Gas analysis was performed using an open-circuit metabolic gas analysis system connected directly to a mass spectrometer (Model Arco2000; ArcoSystem, Chiba, Japan). Room air was pumped through the chambers at a rate of 0.3 L/min. The expired air was directed to an O_2_/CO_2_ analyzer for mass spectrometry. Motor activity was measured using an infrared sensor (NS-AS01; Neuroscience, Inc., Tokyo, Japan) in each chamber.

### Tissue collection

We collected interscapular brown adipose tissue (iBAT) and hypothalamus tissues between 9 and 12 am (3 to 6 h after lights on) from mice 3 weeks after HFD feeding and stored at -70°C until analysis. To collect the hypothalamus, the brain was placed ventral side up in brain matrix (World Precision Instruments, Sarasota, FL, USA), and a 2 mm-thick coronal slice was dissected out by an anterior coronal cut in the middle of the optic tract, just rostral to infundibulum, and a posterior coronal cut, at the posterior border of the mammillary bodies. The slice was then dissected laterally up to the hypothalamic sulcus and dorsally just above the third ventricle. For microdissection of the PVH, the brain was placed ventral side up in the brain matrix (World Precision Instruments, Sarasota, FL), while two 1 mm-thick sagittal slices (right and left side from the centerline of the brain) were dissected. The slices were observed under a stereoscopic microscope, and the VMH was microdissected based on the key visible anatomical structures [24]. All procedures were conducted rapidly on ice to prevent RNA and protein degradation.

### Protein analysis

Total protein from whole tissues was extracted in a buffer (20 mM Tris, pH 7.4, 150 mM sodium chloride, 10 mM EDTA, 100 mM sodium fluoride, 10 mM sodium pyrophosphate, and 1% Nonidet P-40) containing 10 mM sodium orthovanadate, and a protease inhibitor cocktail (Sigma-Aldrich, St. Louis, MO, USA) (n = 6–7). Proteins were quantified and 10 μg of protein was separated by SDS-PAGE. Following SDS-PAGE, proteins were transferred to PVDF membranes (Millipore, Billerica, MA, USA), blocked with 5% milk in Tris-buffered saline containing 0.1% Tween20, and incubated with primary and secondary antisera in Tween20. Antibody binding was visualized using a Chemilumi-One Super (Nacalai Tesque, Inc., Kyoto, Japan).

The antibodies used for the western blot analyses were a rabbit polyclonal Ab against UCP1 (#ab10983, 1: 1000 dilution, Abcam, Eugene, OR, USA), rabbit polyclonal Ab against PGC1α (#13067, 1: 1000 dilution, Biotechnology, Santa Cruz, CA, USA), mouse monoclonal antibody against GAPDH (# 97166; 1: 1000 dilution, Cell Signaling Technology, Danvers, MA, USA), and HRP-conjugated goat anti-rabbit or mouse IgG Ab (# 170–6515, 1: 3000 dilution, Bio-Rad, Hercules, CA, USA). The relative densities of the bands were analyzed using the National Institutes of Health Image software (ImageJ) and normalized to the relative density of the GAPDH bands.

## Pair-feeding

In the pair-feeding experiment, all mice were housed in individual cages and fed an HFD from 6 weeks of age (n = 5–8). We measured the HFD intake of each control mouse daily, and then administered Sim1-CRTCDKO mice with the same amount of HFD consumed by control mice for 6 weeks.

## Blood analysis

We collected blood from mice after 3 weeks of HFD feeding when the body weight differences were significant (n = 6–7). Blood sampling was conducted during the light phase from ad libitum-fed mice 3–6 h after the lights were turned off. Blood was collected by decapitation without anesthesia, and the serum was separated using a refrigerated centrifuge. Blood serum samples were analyzed using the appropriate assay kits for triglycerides (Wako Pure Chemical Industries, Osaka, Japan), glucose (Wako Pure Chemical Industries), and non-esterified free fatty acids (Wako Pure Chemical Industries). Serum insulin and leptin levels were measured using mouse insulin ELISA (Mercodia Uppsala, Sweden) and mouse leptin ELISA (Biovendor Laboratory Medicine, Brno, Czech Republic), respectively.

### Melanotan II (MTII) administration

Twelve-week-old mice fed with NCD were used in the present study (n = 5–7). Before the experiment, the mice were housed individually in cages for at least four days for habituation. After 24 h of food deprivation, mice were intraperitoneally administered with MTII (10 mg/kg) or saline. After 30 min, the mice were fed NC, and then 2 h food intake was measured. Before measuring HFD intake, mice were fed an HFD for at least three days for habituation.

### Surgery

Six-week-old female mice and five-week-old male mice underwent ovariectomy (n = 8–9) or orchiectomy (n = 9) surgery. A midline incision was made in the skin and peritoneum of the lower abdomen under anesthesia. After removing the ovary or orchis, the incision of the skin and peritoneum was closed using sutures. All mice were allowed to recover for a week and then fed an HFD for 6 weeks.

### Quantitative PCR (qPCR)

**Fig 1:**
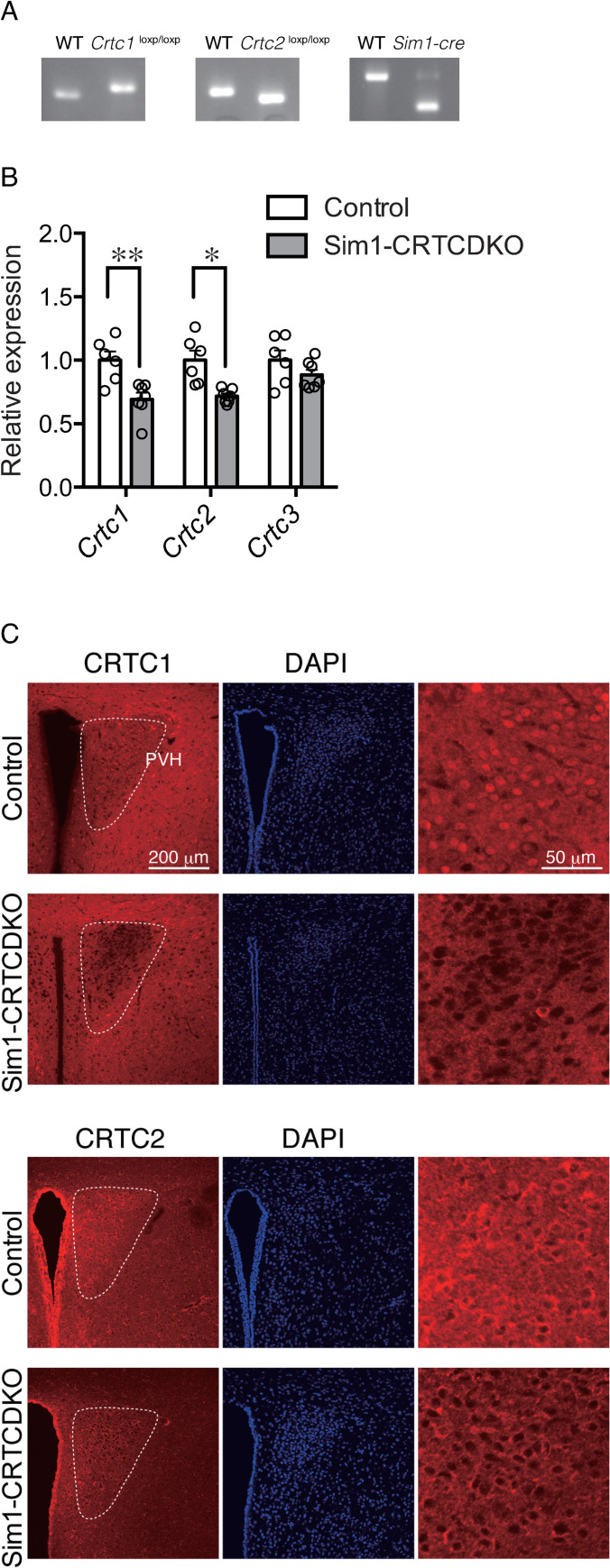
(A) Genotyping result (*Crtc1*^loxP/loxP^, *Crtc2*^loxP/loxP^, and *Sim1*-Cre) of Sim1-CRTCDKO mice. (B) *Crtc1*, *Crtc2*, and *Crtc3* mRNA expression in PVH in control and Sim1-CRTCDKO mice (n = 6–7). Values are presented as mean ± SEM. **: *P* < 0.01, *: *P* < 0.05. (C) Immunohistochemical analysis of CRTC1 and CRTC2 expression in the PVH of Sim1-CRTCDKO mice. Brain slices were stained with anti-CRTC1 antibody (red) or anti-CRTC2 antibody (red) and DAPI (blue).

Total RNA from whole tissues was extracted using TriPure Isolation Reagent (Roche, Mannheim, Germany). cDNA was generated using a PrimeScript™ RT Reagent Kit (Takara Bio, Inc., Shiga, Japan) (n = 6–7, Fig 1B; n = 6–7, Fig 8). cDNA was quantified using a LightCycler 480 instrument (Roche, Indianapolis, IA, USA) and KAPA SYBR FAST qPCR Master Mix 2× optimized for LightCycler 480 (Kapa Biosystems, Wilmington, MA, USA). Gene expression data are presented relative to the parallel measured expression of housekeeping cDNA, *glyceraldehyde-3-phosphate dehydrogenase* (*GAPDH*).

### Primer sequences for qPCR

*Gapdh*: F-5′ AGGTCGGTGTGAACGGATTTG 3′, R-5′ TGTAGACCATGTAGTTGAGGTCA 3’
*Crtc1*: F-5′ AGACAGACAAGACCCTTTCTAAGCA 3′, R-5′ CAGGACTTGGGCCTGGAA 3′
*Crtc2*: F-5′ GGCCTTCGAGGAGGTGAT 3′, R-5′ TATAAGCCAGTCGCAGTTTTTGG 3′
*Crtc3*: F-5′ TGACTCACCTGGGGATAAGAAC 3′, R-5′ GTGGCACTTGAGGGACGAG 3′
*Pacap*: F-5′ ACCATGTGTAGCGGAGCAAG 3′, R-5′ CTGGTCGTAAGCCTCGTCT 3′
*Crf*: F-5′ CCTCAGCCGGTTCTGATCC 3′, R-5′ GCGGAAAAAGTTAGCCGCAG 3′
*Crfbp*: F-5′ ATGTCACCGAACTTCAAACTCC 3′, R-5′ TTCTTGCACCTCTAGGTAGCG 3′
*Sst*: F-5′ CTCTGCATCGTCCTGGCTTT 3′, R-5′AAGTACTTGGCCAGTTCCTGTTT 3′
*Avp*: F-5′ GCCAGGATGCTCAACACTACG 3′, R-5′ TCTCAGCTCCATGTCAGAGATG 3′
*Oxt*: F-5′ GCCAGGAGGAGAACTACCTG 3′, R-5′ CTCCGAGAAGGCAGACTCAG 3′
*Esr1*: F-5′ CCTCCCGCCTTCTACAGGT 3′, R-5′ CACACGGCACAGTAGCGAG 3′
*Esr2*: F-5′ CTGTGCCTCTTCTCACAAGGA 3′, R-5′ TGCTCCAAGGGTAGGATGGAC 3′
*Mc4r*: F-5′ CCCGGACGGAGGATGCTAT 3′, R-5′ TCGCCACGATCACTAGAATGT 3′

### Statistical analyses

Values are presented as the mean ± standard error of the mean (SEM). Statistical analyses were performed using Welch’s t-test for comparisons between the two groups. Brown-Forsythe and Welch analysis of variance (ANOVA) tests were performed to compare more than three groups (Prism 8.0; GraphPad Software Inc., San Diego, CA, USA). When significant differences were found, Dunnett’s T3 multiple comparison test was performed as a post-hoc analysis. A two-way repeated-measures ANOVA was used to compare group differences in body weight gain and food intake. When significant differences were found, Sidak’s multiple comparison test was performed as a post-hoc analysis. When significant differences were not found, Welch’s t-test was performed at each time point. Differences were considered significant at P < 0.05.

## Result

### Generation of mice lacking CRTC1 and CRTC2 specifically in Sim1 cells

Mice lacking CRTC1 and CRTC2 in Sim1 cells (Sim1-CRTCDKO mice) were generated by crossing *Crtc1*^loxP/loxP^; *Crtc2*^loxP/loxP^ mice with *Sim1*-Cre mice, in which Cre recombinase was expressed exclusively in the PVH and some part of the amygdala (Fig 1A). Quantitative PCR analysis of RNA isolated from the micro-dissected PVH showed that both *Crtc1* and *Crtc2* mRNA were significantly reduced in Sim1-CRTCDKO mice compared to control mice (Fig 1B). *Crtc3* mRNA levels in Sim1-CRTCDKO mice were comparable to those in control mice.

We also checked CRTC1 and CRTC2 protein expression by immunohistochemistry (Fig 1C). Both CRTC1 and CRTC2 immunoreactivity was detected in the PVH of control mice. However, CRTC1 immunoreactivity almost completely disappeared in the PVH of Sim1-CRTCDKO mice, although CRTC1 expression surrounding the PVH remained intact. CRTC2 immunoreactivity in the PVH of Sim1-CRTCDKO mice was weaker than that in control mice.

### Body weight change in normal chow diet (NCD) and high fat diet (HFD)

**Fig 2:**
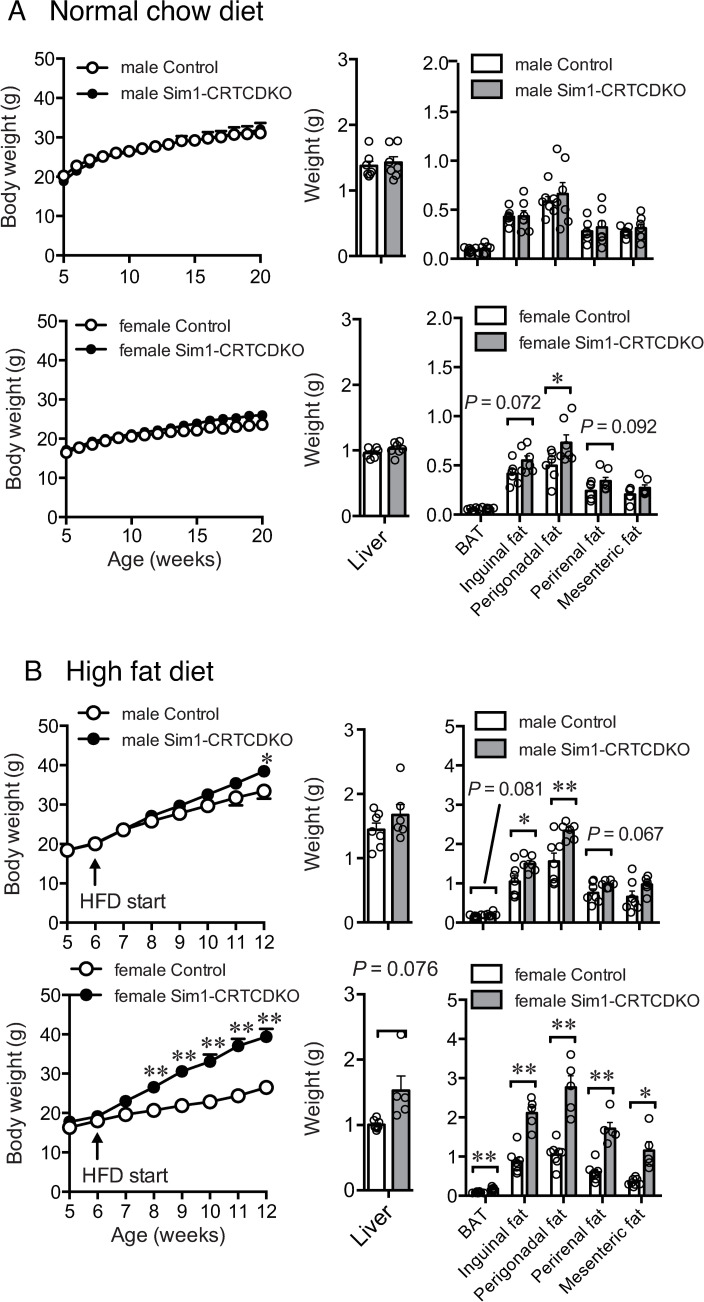
(A) Body weight change (left panel) in control mice and Sim1-CRTCDKO mice in normal chow diet feeding (n = 6–7). Values are presented as mean ± SEM. Tissue weight at 20 weeks of age (right panel). *: *P* < 0.05. (B) Body weight change (left panel) in control mice and Sim1-CRTCDKO mice in HFD feeding (n = 5–8). Values are presented as mean ± SEM. **: *P* < 0.01, *: *P* < 0.05. Tissue weight at 12 weeks of age (right panel). **: *P* < 0.01, *: *P* < 0.05.

In NCD feeding, female Sim1-CRTCDKO mice showed slight but significant body weight gain (*P* < 0.0001; time × two groups interaction in two-way repeated measures ANOVA). Perigonadal adipose tissue size was significantly increased in female Sim1-CRTCDKO mice compared to the control group at 20 weeks of age (Fig 2A, right). There was no significant difference in body weight gain and tissue size between male Sim1-CRTCDKO mice and control mice fed normal chow.

In HFD feeding, both female and male Sim1-CRTCDKO mice showed significant body weight gain with significantly increased adipose tissue size (Fig 2B). The obese phenotype of female Sim1-CRTCDKO mice was stronger than that of male mice; therefore, female mice were mainly used in subsequent experiments.

### Food intake of NCD and HFD

**Fig 3:**
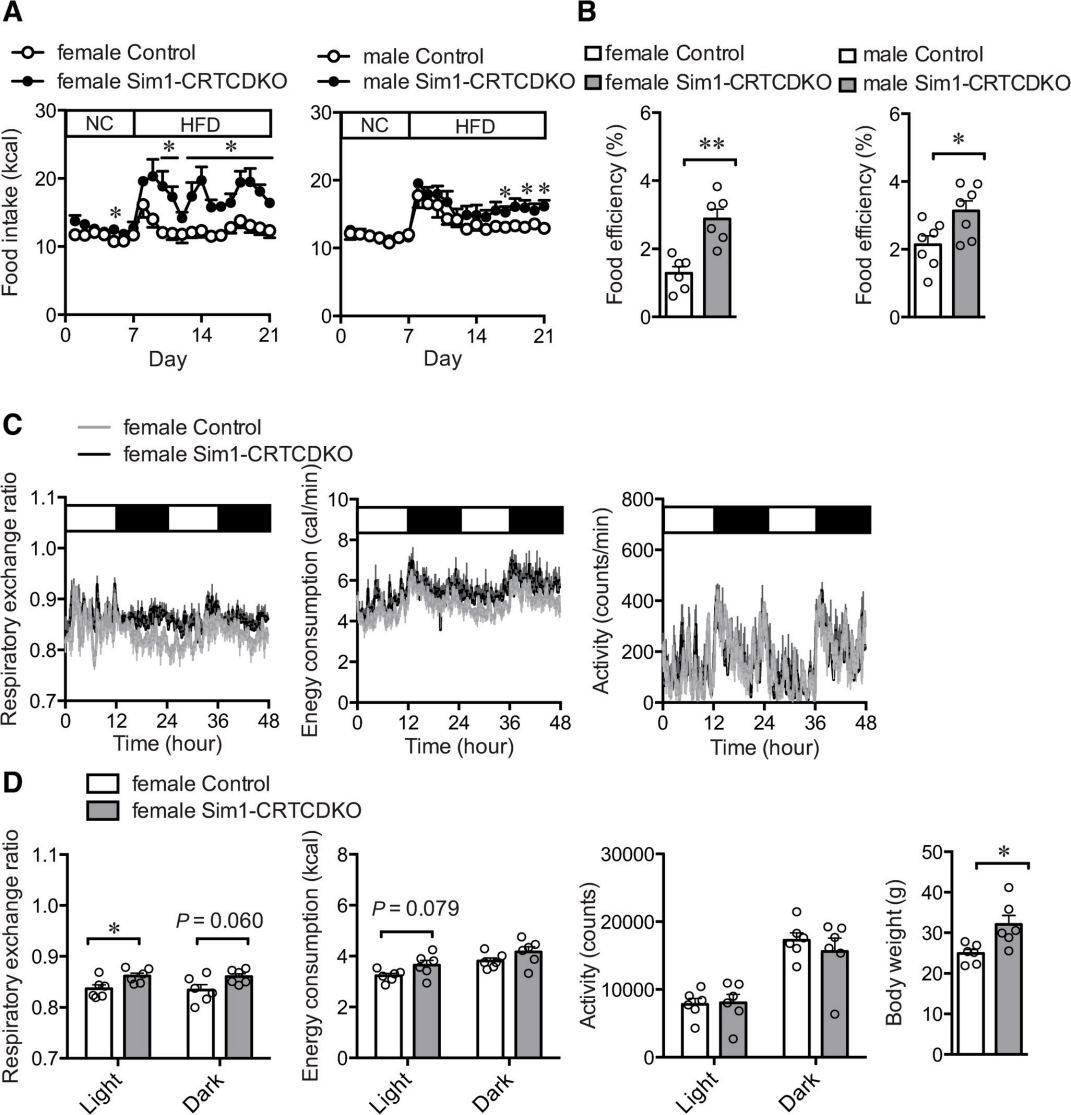
(A) Daily food intake. From day 1 to day 7, all mice were fed normal chow diet. From day 8 to day 21, mice were fed HFD (n = 6–7). Values are presented as mean ± SEM. **: *P* < 0.01, *: *P* < 0.05. (B) Food efficiency calculated from body weight gain and total caloric intake (from day 8 to day 21). Values are presented as mean ± SEM. **: *P* < 0.01, *: *P* < 0.05. (C) Respiratory exchange ratio (RER), energy consumption, and motor activity of control mice and Sim1-CRTCDKO mice after 4 weeks of HFD feeding (n = 6). Values are presented as mean ± SEM. (D) Average RER, cumulative energy consumption, cumulative motor activity for 24 h (light and dark phases), and body weight at the day of respiratory gas measurement. *: *P* < 0.05.

NCD intake in the first 7 days (Day 1 to Day 7) was slightly higher in female Sim1-CRTCDKO mice than in control mice. HFD intake was significantly increased in Sim1-CRTCDKO mice (from day 8 to day 21) (Fig 3A).

Male Sim1-CRTCDKO mice consumed comparable amounts of normal chow compared to control mice (from day 1 to day 7). However, HFD intake was significantly increased in male Sim1-CRTCDKO mice compared to that in control mice (from day 8 to day 21).

Food efficiency calculated from total caloric intake (from day 8 to day 21) and body weight gain in the same period was significantly increased in both female and male Sim1-CRTCDKO mice; however, the difference in food efficiency compared to control mice was more prominent in female Sim1-CRTCDKO mice than in male mice (Fig 3B).

### Respiratory gas analysis

We conducted respiratory gas analysis after 4 weeks of HFD feeding when the body weight of female Sim1-CRTCDKO mice was significantly higher than that of control mice (Fig 3D, right). The respiratory exchange ratio (RER) of female Sim1-CRTCDKO mice was slightly higher throughout the day (Fig 3C), and the average RER in female Sim1-CRTCDKO mice was significantly higher in the light phase (Fig 3D, left). There was no significant difference in energy consumption and motor activity between Sim1-CRTCDKO and control mice.

### Two diet choice experiment, AIN-93M vs. HFD

**Fig 4:**
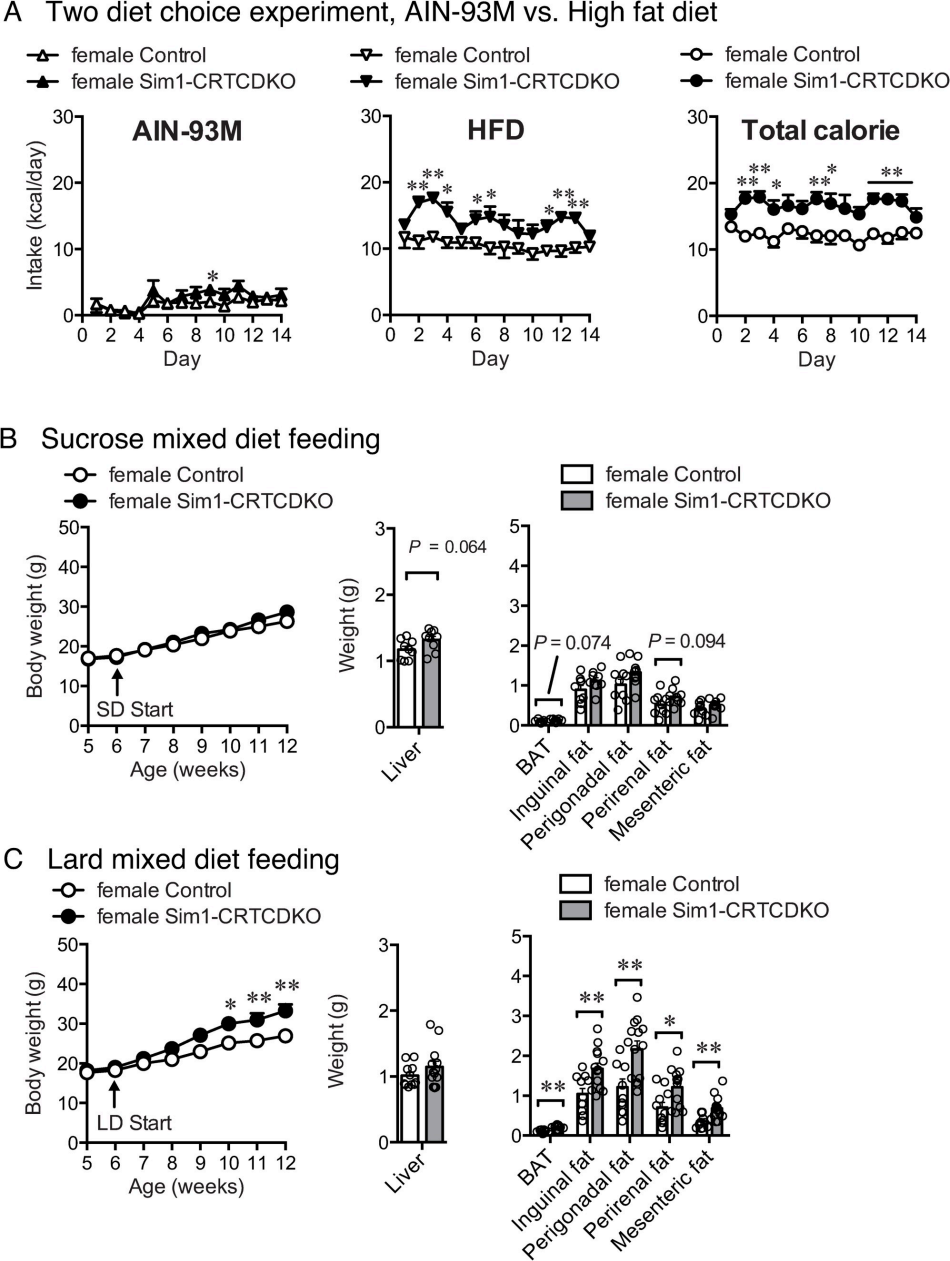
(A) Daily AIN-93M, HFD, and total caloric intake. Mice were fed both AIN-93M and HFD ad libitum (n = 5–6). Values are presented as mean ± SEM. **: *P* < 0.01, *: *P* < 0.05. (B) Body weight change (left panel) in control mice and Sim1-CRTCDKO mice in SD feeding (n = 9). Values are presented as mean ± SEM. Tissue weight at 12 weeks of age (right panel). (C) Body weight change (left panel) in control mice and Sim1-CRTCDKO mice in LD feeding (n = 11–13). Values are presented as mean ± SEM. **: *P* < 0.01, *: *P* < 0.05. Tissue weight at 12 weeks of age (right panel). **: *P* < 0.01, *: *P* < 0.05.

To check whether the deficiency of CRTCs affects food palatability, we conducted two diet choice experiments. We administered AIN-93M standard diet and HFD simultaneously to mice and measured the intake of each diet. Both Sim1-CRTCDKO and control mice consumed more HFD than the AIN-93M mice (Fig 4A). The intake of both HFD and AIN-93M was increased in Sim1-CRTCDKO mice. Consequently, the ratio of HFD intake and AIN-93M in Sim1-CRTCDKO mice was comparable to that in control mice.

### Body weight change in standard diet (SD) and lard diet (LD)

HFD (#D12492, Research Diet) contains lard and sucrose, and both ingredients may increase palatability and possibly daily intake. To check whether lard or sucrose may be attributed to the increase in body weight gain in female Sim1-CRTCDKO mice, we measured body weight gain by using a 20% sucrose mixed diet (SD) or a 20% lard mixed diet (LD).

SD feeding did not increase body weight gain in female Sim1-CRTCDKO mice compared to control mice, and there was no significant difference in body weight and tissue weight between Sim1-CRTCDKO mice and control mice (Fig 4B left). In contrast, female Sim1-CRTCDKO mice showed significant body weight gain in LD feeding, and after 4 weeks of LD feeding, adipose tissue weight was significantly increased in female Sim1-CRTCDKO mice compared to that in control mice (Fig 4D).

### Protein analysis in brown adipose tissue

**Fig 5:**
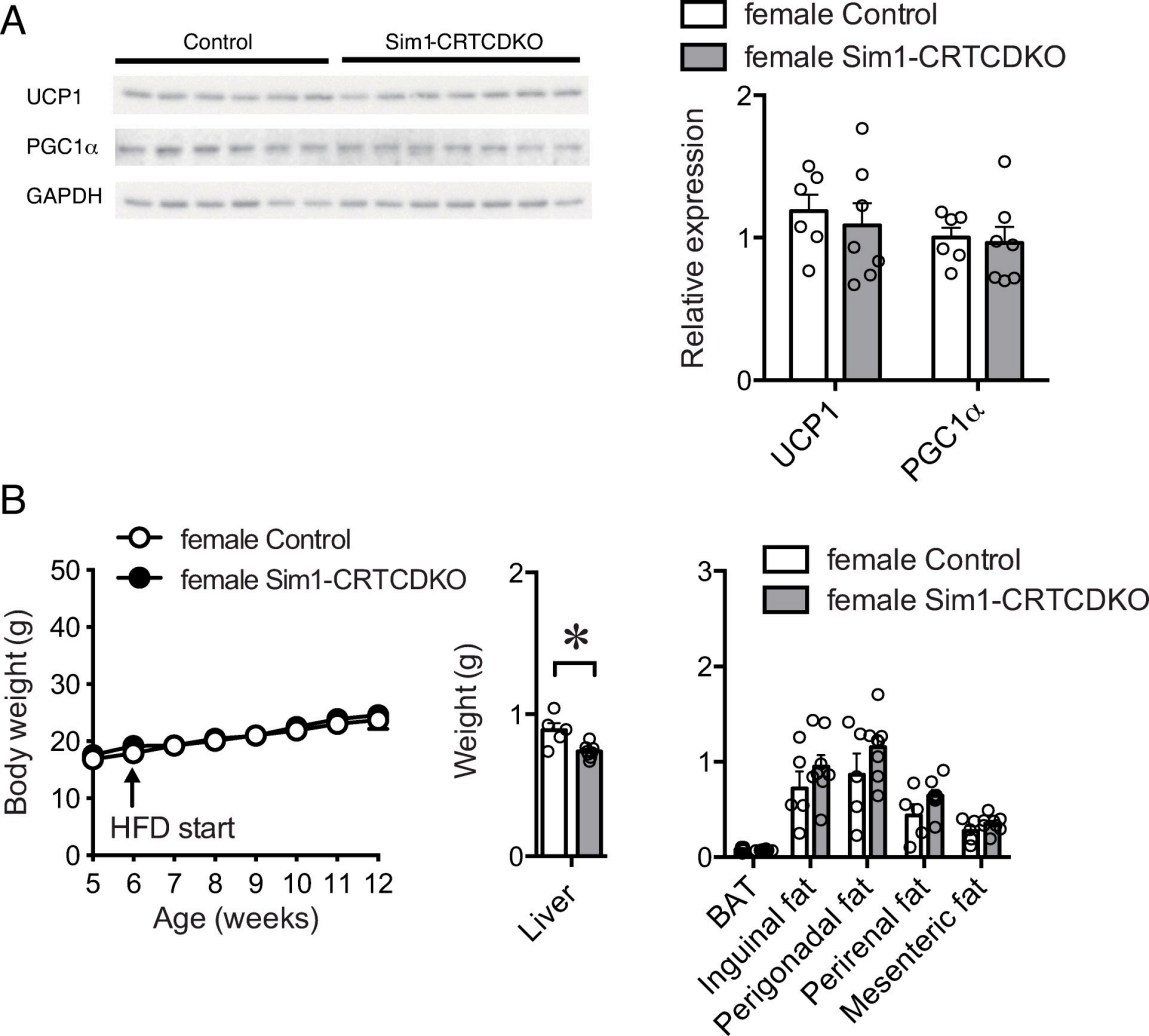
(A) Western blot analysis of protein expression in iBAT from mice fed a HFD for 3 weeks (n = 6–7). (B) Body weight change (left panel) in control mice and Sim1-CRTCDKO mice in pair-feeding (n = 5–8). Values are presented as mean ± SEM. Sim1-CRTCDKO mice were administered the same amount of HFD consumed by control mice daily. Pair-feeding conducted for 6 weeks (6 to 12 weeks of age). Tissue weight at 12 weeks of age (right panel). *: *P* < 0.05.

We next investigated whether Sim1 cell-specific CRTC1 and CRTC2 deficiency affected protein expression related to energy expenditure in brown adipose tissue. After 3 weeks of HFD feeding, we collected adipose tissue and analyzed protein expression. Protein expression of uncoupling protein 1 (UCP1) and peroxisome proliferator-activated receptor gamma coactivator 1α (PGC1α) in the brown adipose tissue of female Sim1-CRTCDKO mice was comparable to that in the control mice (Fig 5A).

### Pair-feeding

Since HFD intake was increased in Sim1-CRTCDKO mice, energy expenditure did not change in Sim1-CRTCDKO mice, and it is speculated that the significant body weight gain seen in Fig 2 might be mainly attributed to the increase in HFD intake by female Sim1-CRTCDKO mice. Therefore, we conducted a pair-feeding experiment in which both Sim1-CRTCDKO mice and control mice were given the same amount of food.

Pair-feeding blunted the difference in body weight gain in the HFD between female Sim1-CRTCDKO mice and control mice (Fig 5B, left). There was no significant difference in body weight gain and adipose tissue size between the control and female Sim1-CRTCDKO mice fed the HFD until the end of the experiment (Fig 5B, right).

### Blood analysis

**Fig 6:**
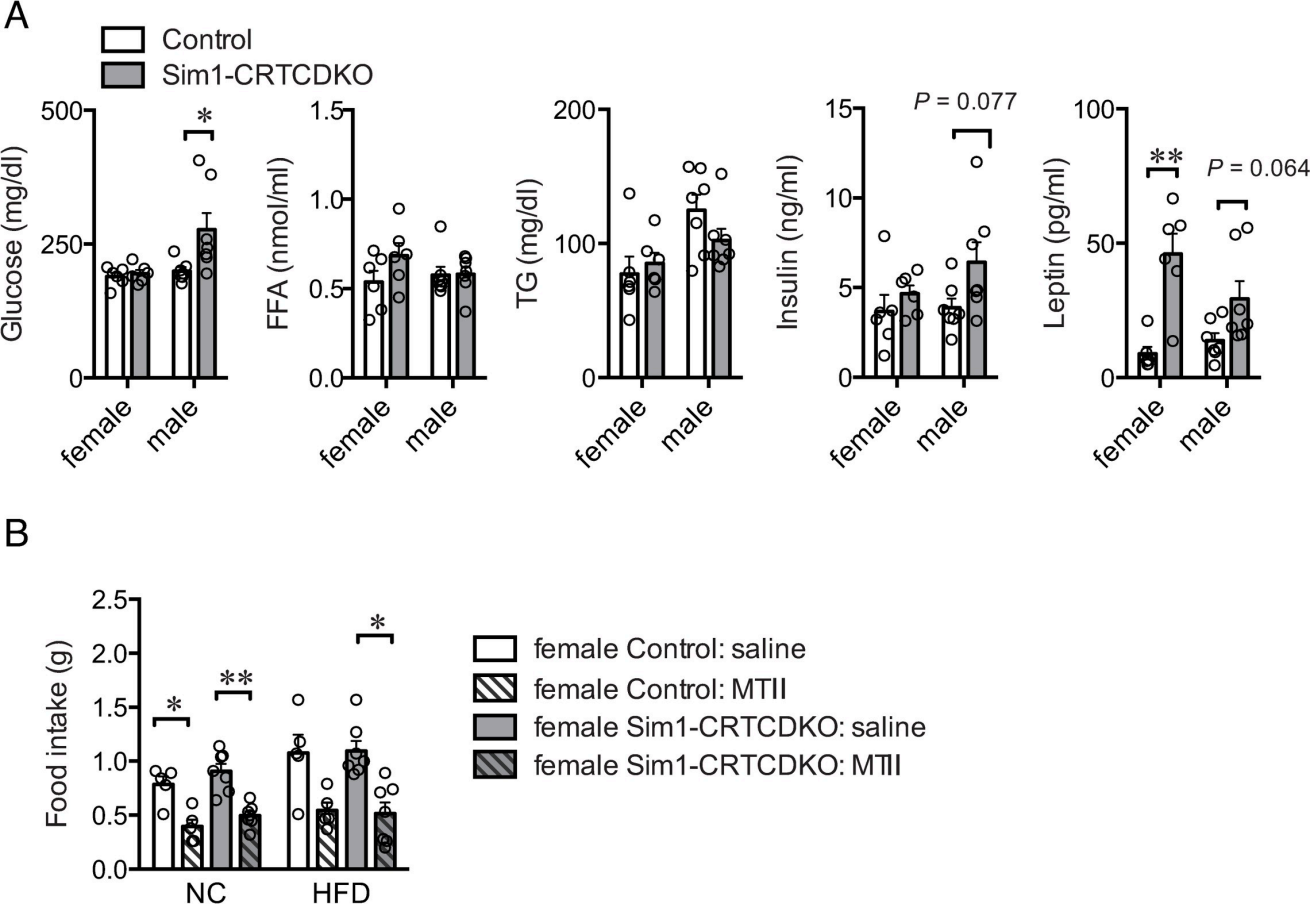
(A) Serum triglyceride (TG), glucose, free fatty acids (FFA), insulin, and leptin of control mice and Sim1-CRTCDKO mice after 3 weeks of HFD feeding (n = 6–7). Values are presented as mean ± SEM. **: *P* < 0.01, *: *P* < 0.05. (B) Response to MC3/4R agonist on food intake. After 24 h of fasting, mice were administered saline or MTII intraperitoneally. After 30 min, mice were fed a NC or HFD, and then food intake for 2 h was measured (n = 5–7). Values are presented as mean ± SEM. **: *P* < 0.01, *: *P* < 0.05.

After 3 weeks of HFD feeding, blood was collected, and data were analyzed. Free fatty acid and triglyceride levels in female and male Sim1-CRTCDKO mice were comparable to those in the control mice (Fig 6A). Glucose levels were significantly higher in male Sim1-CRTCDKO mice than in control mice. Insulin levels tended to be higher in the male Sim1-CRTCDKO mice. Leptin levels were significantly higher in both male and female Sim1-CRTCDKO mice than in control mice.

### Response to MC3/4R agonist MTII on food intake

MC4R plays an important role in the regulation of appetite in PVH cells [19,25]. In addition, it is likely that the MC4R agonist activates both CRTC1 and CRTC2 because MC4R is coupled with Gs. Therefore, we examined the effect of the MC3/4R agonist (Melanotan II: MTII) on food intake. After 24 h of fasting, we injected MTII intraperitoneally and then measured NC or HFD intake. MTII remarkably reduced food intake in both the groups (Fig 6B). However, there was no significant difference in food intake between the control and female Sim1-CRTCDKO mice.

### Body weight change of ovariectomized (OVX) or orchiectomized (ORX) mice in HFD feeding

The obese phenotype and hyperphagic phenotype in female Sim1-CRTCDKO mice was stronger than that in male mice (Figs 2 and 3). The sex difference in the phenotype observed in the current study might be attributed to sex hormones. Therefore, we surgically removed ovaries and testes from Sim1-CRTCDKO and control mice. After one-week recovery period, all mice were fed a HFD, and body weight was measured.

**Fig 7:**
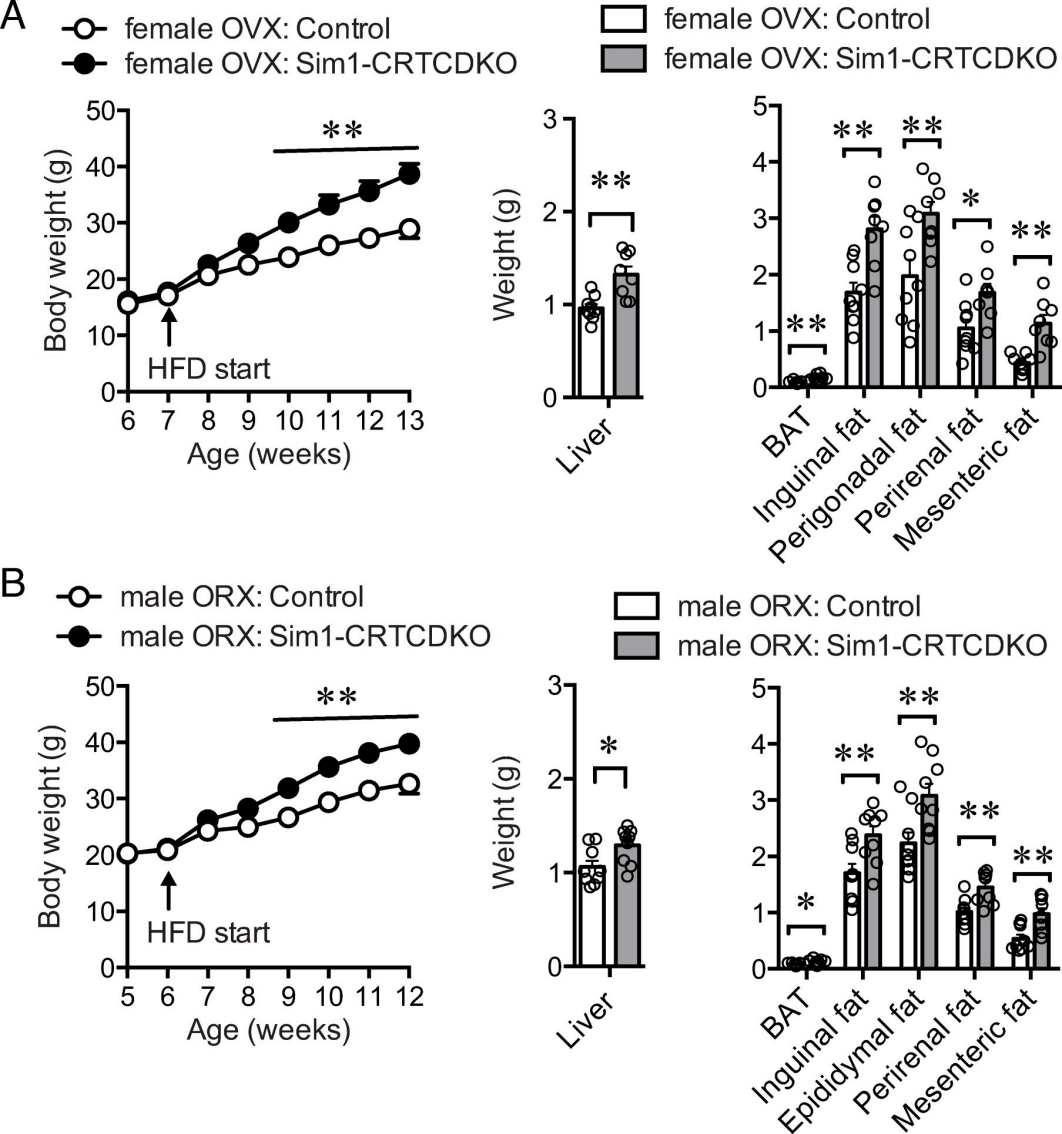
(A) Body weight change (left panel) in ovariectomized control mice and Sim1-CRTCDKO mice in HFD feeding (n = 8–9). Six-week-old mice were ovariectomized, and started HFD feeding after a week. Values are presented as mean ± SEM. **: *P* < 0.01. Tissue weight at 12 weeks of age (right panel). **: *P* < 0.01, *: *P* < 0.05. (B) Body weight change (left panel) in orchiectomized control mice and Sim1-CRTCDKO mice in HFD feeding (n = 9). Five-week-old mice were orchiectomized, and started HFD feeding after a week. Values are presented as mean ± SEM. **: *P* < 0.01. Tissue weight at 12 weeks of age (right panel). **: *P* < 0.01, *: *P* < 0.05.

Both OVX and ORX mildly increased body weight and tissue size in both Sim1-CRTCDKO mice and control mice compared to those in intact mice (Figs 2 and 7). If sex hormones released from the ovaries or testes play an important role in the significant weight gain seen in Sim1-CRTCDKO mice, OVX or ORX would blunt the body weight difference between Sim1-CRTCDKO mice and control mice. Female Sim1-CRTCDKO mice showed significant body weight gain compared to control mice, as shown in Fig 2. However, female control mice gained more body weight than intact mice (Fig 2). As a result, the differences in body weight gain between control mice and Sim1-CRTCDKO mice were reduced after surgery. Male Sim1-CRTCDKO mice gained more body weight than intact mice (Fig 2). As a result, the differences in body weight gain between control mice and Sim1-CRTCDKO mice were more prominent after surgery.

### Quantitative PCR analysis of hypothalamus sample

**Fig 8:**
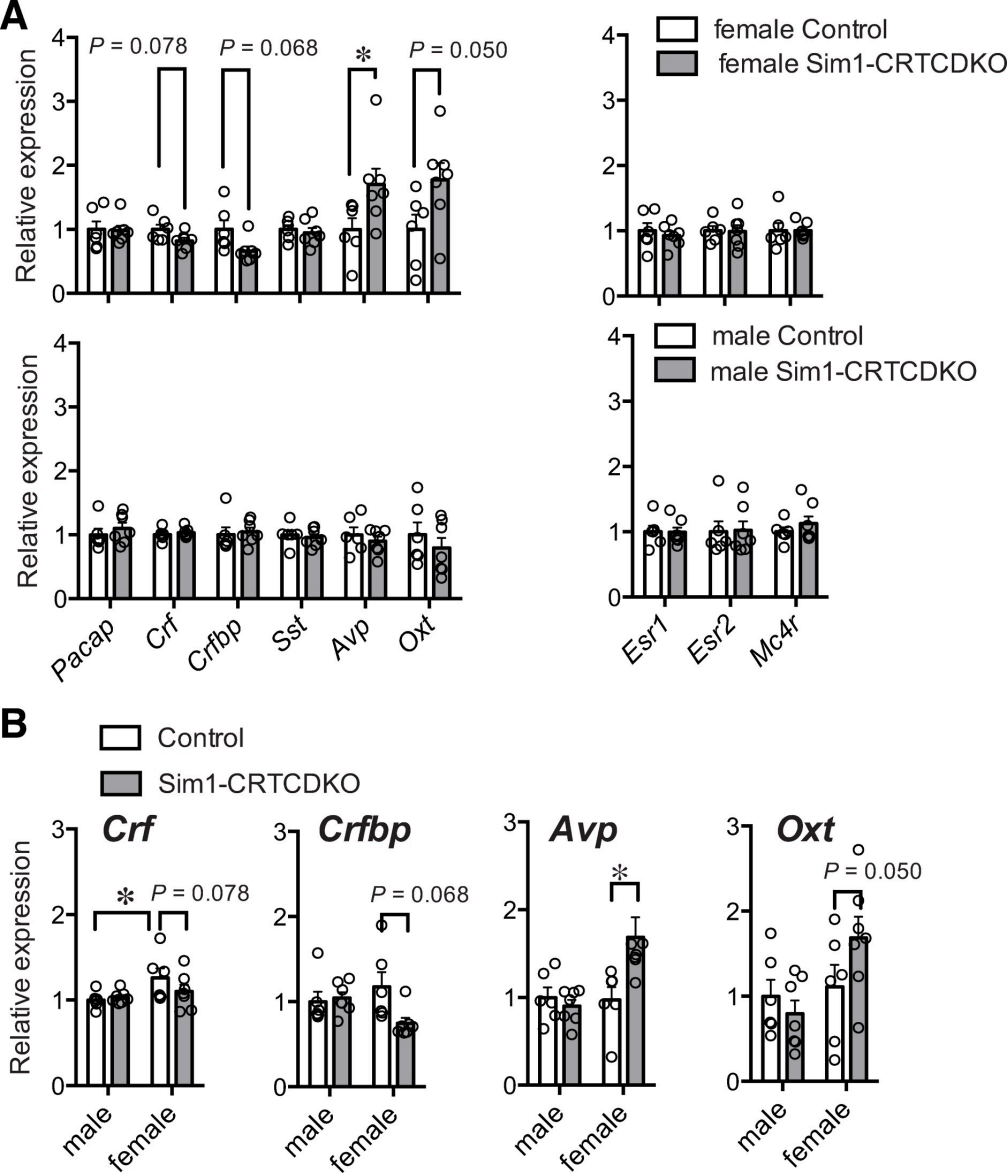
(A) mRNA expression in the hypothalamus from mice fed a HFD for 3 weeks (n = 6–7). The mRNA of neurotransmitters (left panel) and receptors (right panel) expressed in the PVH were measured. Values are presented as mean ± SEM. *: *P* < 0.05 (t-test). (B) Sex differences in mRNA expression. Values are presented as mean ± SEM. *: *P* < 0.05 (t-test).

Both CRTC1 and CRTC2 are coactivators of the transcription factor CREB. Therefore, we speculated that CRTC deficiency may affect CREB target genes or downstream genes of CREB target genes. Therefore, we collected hypothalamus from mice fed HFD for 3 weeks since only HFD-fed mice showed a significant phenotype, and checked the mRNA expression of PVH-specific genes. The mRNA expression of corticotropin-releasing factor (*Crf*) and corticotropin-releasing factor binding protein (*Crfbp*) were slightly decreased, arginine vasopressin (*Avp*) was significantly increased, and oxytocin (*Oxt*) was slightly increased in female Sim1-CRTCDKO mice compared to that in control mice (Fig 8). In contrast, there was no significant change in the mRNA expression of the PVH-specific gene between male Sim1-CRTCDKO and male control mice. We also compared mRNA expression between males and females (Fig 8B). In control mice, *Crf* expression was significantly higher in female mice than in male mice.

## Discussion

PVH is one of the most important nuclei related to energy metabolism in the hypothalamus. Our current study revealed that CRTCs in the PVH play an important role in appetite and body weight regulation, especially under HFD feeding. Both the energy consumption and thermogenesis-related gene expression of iBAT in Sim1-CRTCDKO mice was comparable to that of control mice, suggesting that CRTC deficiency might not affect energy expenditure. Furthermore, pair-feeding abolished the difference in body weight gain between Sim1-CRTCDKO mice and control mice fed an HFD. These results suggest that the significant body weight gain observed in Sim1-CRTCDKO mice may be caused by hyperphagia, not by reduced energy expenditure.

Under NCD feeding, only female Sim1-CRTCDKO mice showed slight but significant body weight gain (p < 0.05, two-way repeated ANOVA), and male Sim1-CRTCDKO mice did not show significant body weight gain compared to control mice. Under HFD feeding, although both male and female Sim1-CRTCDKO mice showed significant body weight gain, the difference in body weight between Sim1-CRTCDKO mice and control mice was more prominent in female Sim1-CRTCDKO mice than in male mice. We hypothesized that sex differences in body weight gain might be caused by sex hormones. Therefore, we performed OVX and ORX surgeries. OVX slightly reduced the difference in body weight between Sim1-CRTCDKO mice and control mice in the HFD group. In contrast, ORX accelerated the body weight gain in Sim1-CRTCDKO mice, making the difference in body weight gain between groups more prominent. These results indicate that CRTCs in Sim1 cells may be involved in the suppressive effect of female sex hormones. On the other hand, male sex hormones may suppress HFD intake when CRTCs are deficient or impaired in Sim1 cells. It has been reported that CRF expression levels are higher in female rats than in male rats at 60 days old [26]. We also found that *Crf* mRNA expression was higher in female mice than in male mice. In addition, stress-induced CRF expression was also high in female mice [27]. Therefore, sex differences in CRF expression or sensitivity to stress in PVH neuronal cells may cause sex differences in the sensitivity to HFD between control mice and Sim1-CRTCDKO mice. Further studies should reveal the precise mechanism that causes sex differences due to the loss of CRTCs in Sim1 cells.

Sim1-CRTCDKO mice showed significant body weight gain only in HFD feeding, not in NCD feeding, suggesting that CRTCs in Sim1 cells may regulate the motivation to eat HFD (preference) or dietary fat-induced satiety. In the two-diet choice experiment, Sim1-CRTCDKO mice consumed HFD and AIN93M in almost the same ratio as control mice, indicating that PVH CRTCs may not regulate the motivation to eat HFD. The addition of lard (dietary fat) to the AIN93 standard diet induced obesity in Sim1-CRTCDKO mice, indicating that CRTCs in Sim1 cells play an important role in the regulation of dietary fat-induced satiety or dietary fat-induced suppression of food intake.

In the hypothalamus, *Crf* and *Crfbp* mRNA levels tended to be lower in Sim1-CRTCDKO mice than in control mice. This result coincides with a previous study reporting that CRTC2 regulates CRF expression in the PVH [28]. Zhu et al. reported that postnatal manipulation of CRF neuronal responsiveness accelerates HFD-induced obesity [29]. In addition, Okamoto et al. reported that selective activation of CRF neurons by AMP-activated protein kinase in PVH-stimulated high-carbohydrate diet intake and reduced HFD intake [30]. These reports partially coincide with our current results. Thus, it may be possible that CRTCs in the PVH regulate HFD intake by regulating CRF expression.

We also observed that *Oxt* and *Avp* mRNA levels were higher in Sim1-CRTCDKO mice fed with HFD than in control mice. Mice lacking OXT in the PVH are more sensitive to HFD-induced obesity due to reduced energy expenditure [31], and pharmacogenetic activation of OXT neurons in the PVH increases energy expenditure in mice [32]. AVP neurons in the PVH are activated by MTII and the direct activation of AVP neurons in the PVH suppresses food intake in mice [33]. Since both OXT and AVP have suppressive effects on food intake, the hyperphagia observed in Sim1-CRTCDKO mice may not be caused by the upregulation of *Oxt* and *Avp* mRNA levels. Therefore, the increase in *Oxt* and *Avp* mRNA observed in female Sim1-CRTCDKO mice may indicate the impairment of the suppressive effect on appetite by OXT and AVP. Although the precise mechanism that increases OXT and AVP expression by CRTC deficiency remains unknown, the increase in OXT and AVP expression may be a homeostatic adaptation to HFD or obesity. The impairment of the suppressive effect on appetite by OXT and AVP may cause a hyperphagic phenotype in Sim1-CRTCDKO mice. Further studies should reveal how CRTCs in Sim1 cells regulate HFD intake.

We found hyperleptinemia in female Sim1-CRTCDKO mice fed a HFD. Leptin stimulates proopiomelanocortin (POMC) expression in arcuate nucleus neurons through signal transducer and activator of transcription 3 activation. One of the POMC-derived peptides, alpha-melanocyte stimulating hormone (αMSH), binds to MC4R in PVH neurons and strongly suppresses food intake [25]. We speculated that Leptin/αMSH/MC4R/CRTC signaling in PVH plays an important role in appetite regulation, we investigated the effect of the MC4R agonist, melanotan-II (MTII), on food intake. However, MTII significantly reduced food intake in Sim1-CRTCDKO mice, similar to that observed in control mice. These results indicate that CRTCs in the PVH are not involved in the acute suppression of food intake by MC4R.

Both CRTC1 and CRTC2 are activated by cAMP and Ca^2+^, respectively. It has been reported that G_s_ knockout in Sim1 cells causes obesity due to reduced energy expenditure but does not change food intake [34]. In contrast, G_q_ knockout in Sim1 cells also causes obesity owing to hyperphagia, but does not change energy expenditure [35]. These results indicate that cAMP and Ca^2+^ signaling in Sim1 cells have a distinct role in body weight regulation, cAMP signaling is more related to energy expenditure, and Ca^2+^ signaling is related to appetite. In this study, we observed hyperphagia and no change in energy expenditure in Sim1-CRTCDKO mice, indicating that Ca^2+^ signaling may activate CRTC-regulating gene expression related to appetite suppression. However, further studies are required to elucidate this point.

Franck et al. reported that CREB knockout, specifically in Sim1 cells, causes obesity due to reduced energy expenditure [36]. Although CRTC is a coactivator of CREB and CREB was deleted in the same *Sim1*-cre mice, our Sim1-CRTCDKO mice did not show the same phenotype in terms of energy expenditure. CREB knockout sometimes compensates for other transcription factors [37], such as activating transcription factor-1 (ATF1) or cAMP response element modulator (CREM), since these transcription factors partially share a DNA binding site [38]. Therefore, compensation by ATF1 or CREM may cause a difference between CRTC deficiency and CREB deficiency in PVH.

In addition to PVH, Sim1 is expressed in the supraoptic nucleus, medial amygdala (MeA), and nucleus of the lateral olfactory tract [19]. Both CRTC1 and CRTC2 are also expressed in the same brain region [11]. Although PVH is a major brain region that regulates appetite in the brain [12,25], recent evidence has revealed that MeA is also involved in the regulation of appetite [39]. Therefore, it is possible that the loss of CRTCs in MeA might cause hyperphagia and obesity in Sim1-CRTCDKO mice. Further studies should reveal which region (PVH or MeA) of CRTCs is involved in the regulation of HFD intake.

In conclusion, our current study revealed that CRTCs in the PVH play an important role in appetite regulation. Since the hyperphagic phenotype was more evident in HFD feeding, CRTCs in PVH may respond to HFD feeding, inducing downstream target genes and suppressing appetite. We found that AVP and OXT expression was increased in the hypothalamus of Sim1-CRTCDKO mice, indicating that CRTCs may indirectly regulate AVP and OXT expression. We also found that CRF expression was slightly decreased in the hypothalamus of Sim1-CRTCDKO mice. Further studies are needed to elucidate whether these changes in neuropeptide expression cause obesity.

## Supporting information

S1 Fig (TIF)
