# Supplementary figures and images for: Disruption of CRTC1 and CRTC2 in Sim1 cells strongly increases high-fat diet intake in female mice but has a modest impact on male mice
Source: PLoS One. 2022 Jan 12;17(1):e0262577. doi: 10.1371/journal.pone.0262577 (PMC8754333; doi:10.1371/journal.pone.0262577)

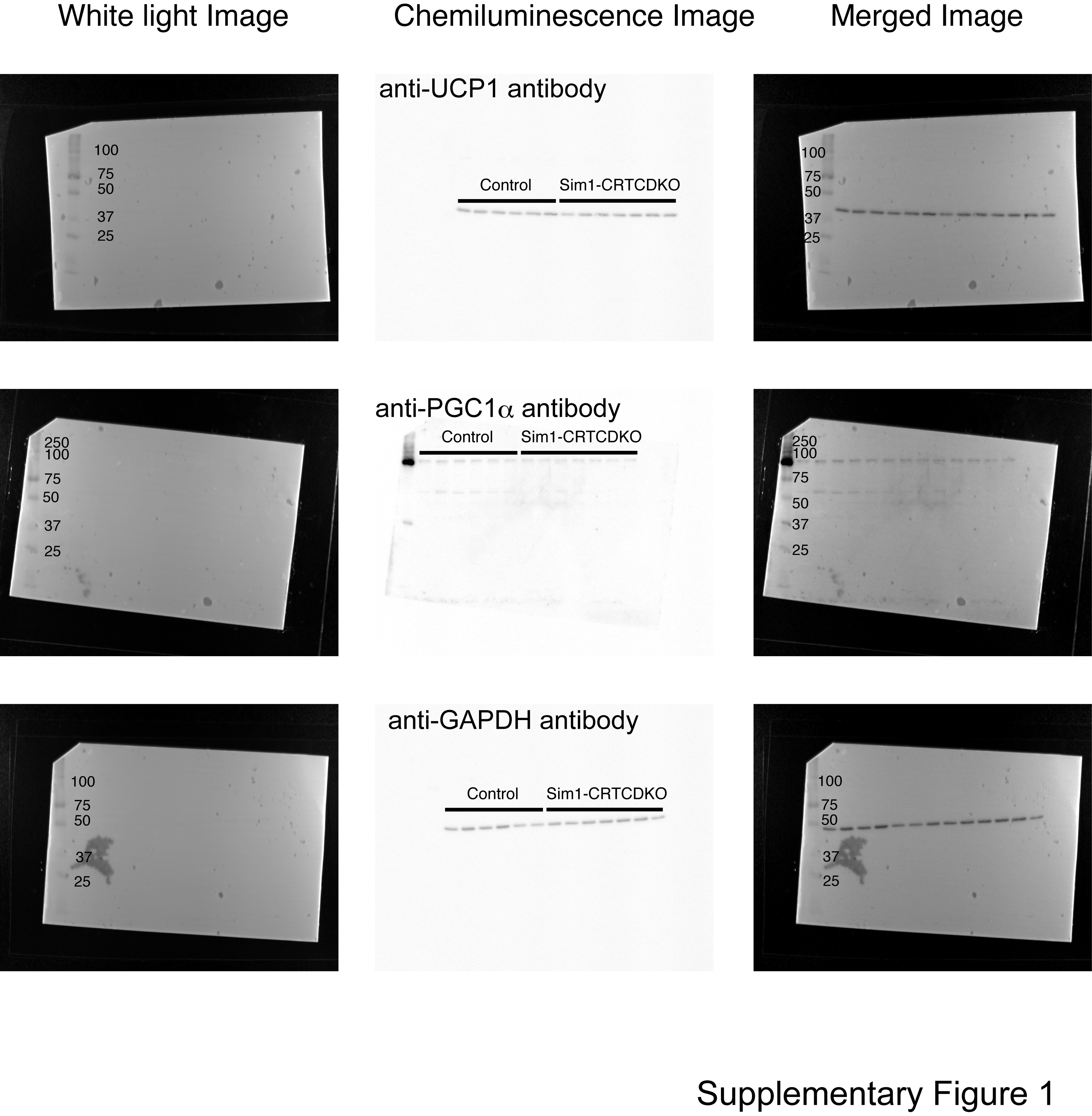

Supplement: S1 Fig — (TIF) [file pone.0262577.s001.tif]
